# Central motor commands to human elbow joint muscles during simultaneous changes in their force and length: a preliminary study

**DOI:** 10.3389/fphys.2026.1743336

**Published:** 2026-02-05

**Authors:** Andriy Gorkovenko, Elena Kolosova, Dmytro Shushuiev, Andriy Maznychenko, Alexander Kostyukov

**Affiliations:** Department of Movement Physiology, Bogomoletz Institute of Physiology, National Academy of Sciences of Ukraine, Kyiv, Ukraine

**Keywords:** central motor commands, electromyogram, muscle hysteresis, muscle synergy, single-joint movements

## Abstract

**Introduction:**

Muscle hysteresis reflects nonlinear, history-dependent relationships between electromyographic (EMG) activity, muscle length, and force. Although hysteresis has been examined under fixed-length or fixed-force conditions, it remains unclear how EMG hysteresis behaves when muscle length and force change simultaneously, especially when their temporal patterns coincide or oppose each other. This preliminary study aimed to determine how a combination of simultaneous changes in the muscle force and length may influence the EMG hysteresis in elbow flexors.

**Methods:**

A robotic mechatronic device generated cyclic elbow movements while participants produced voluntary forces controlled through visual biofeedback. EMG hysteresis was quantified across various combinations of identical temporal patterns of the muscle length and force changes in form of a double trapezoid. Two patterns of the length changes were of the same amplitude range differing by direction of the movement phases: lengthening–shortening and shortening–lengthening. Each of the above length patterns were combined with five patterns of force change: two maximal amplitude of change, two half maximal amplitude, and one isotonic.

**Results:**

EMG hysteresis was shaped not only by the direction of muscle length change but also strongly by the direction of the accompanying force change. Under isotonic conditions, EMG intensity increased during lengthening and decreased during shortening. With increasing the force amplitude changes in *coinciding* direction, the amplitude of EMG hysteresis, i.e., its difference between shortening and lengthening branches, increases, whereas with force changes in the *opposing* direction, it decreases significantly, even reaching negative values, thereby disrupting the usual direction of hysteresis effects.

**Conclusion:**

This study systematically demonstrates that muscle hysteresis can be significantly dependent on a combination of changes in muscle strength and length, as well as their history. The results open new perspectives for analyzing various problems in human motor control and movement rehabilitation.

## Introduction

The muscles of the human limbs, when performing purposeful movements, interact with external loads and also with each other through internal force transmission. Even under minimal external influence, force interactions between synergistic muscles contribute to shaping the overall motor output. To conceptualize these interactions, muscles are often grouped into functional units or “synergies” with similar mechanical, contractile, or activation properties (Pham et al., 2023; Mulla and Keir, 2023). Three commonly described synergy types include kinematic synergies, which reflect correlations between joint angle changes (Santello and Soechting, 2000; Thakur et al., 2008; Soechting and Flanders, 1997), force (kinetic) synergies involved in generating voluntary hand forces (Santello and Soechting, 2000; Grinyagin et al., 2005), and activation synergies, which represent consistent patterns of muscle co-activation across static postures or dynamic actions (Weiss and Flanders, 2004; Castellini and van der Smagt, 2013; Valero-Cuevas, 2016; Latash et al., 2007). Broader methodological and theoretical perspectives on synergy analysis are summarized by Valero-Cuevas (2016) and Bruton and O’Dwyer (2018).

Scientific validity of the EMG measurement approach is essential, as validated measurements are a prerequisite for the accurate interpretation, reproducibility, and clinical relevance of neurophysiological data. In the study by Santoboni et al., which primarily focuses on the validity and correlative value of ultrasonography and elastosonography in detecting plantar fascia alterations, the EMG component served as a standardized and validated method for characterizing the extent of neurological complications in the study participants (Santoboni et al., 2023). Establishing adequate test-retest reliability is a prerequisite for defining meaningful cut-off values and for ensuring that clinical tests can be used confidently in both cross-sectional and longitudinal assessments (Vitiello et al., 2024). The rigid selection criteria and systematic bias assessment support the conclusion that intervention effects were derived from studies with robust designs and appropriate statistical foundations, thereby underscoring the importance of rigorous statistical planning in clinical research. The systematic review by Migliorini et al. on the pharmacological management of secondary chronic spinal cord injury demonstrates a highly rigorous and structured approach to intervention evaluation and ensures strong methodological and statistical rigor through strict inclusion criteria and comprehensive quality assessment (Migliorini et al., 2024a).

From a physiological viewpoint, skeletal muscle can be considered a nonlinear dynamical system defined by three state variables: muscle length (L), muscle force (F), and activation level (E). Classical studies have typically examined force as a function of length and activation, F(L, E), using ramp-and-hold or triangular length changes at constant stimulation rates. These experiments demonstrated pronounced nonlinearities, including the well-known movement history-dependent hysteresis, where muscles generate higher forces during lengthening than during shortening, and these differences persist after movement termination (Kostyukov and Korchak, 1998; Proske et al., 2014; Fukutani et al., 2019). Similar nonlinearities emerge when assessing L(F, E) relationships under isotonic conditions. Hysteresis loops reflect intrinsic contractile properties and occur even at very low movement speeds, emphasizing the importance of prior movement history (Kostyukov, 1987; Morgan, 1994; Fukutani et al., 2019; de Campos et al., 2022; Pinnell et al., 2019). Most prior investigations analyzed these dependencies by varying only one variable while holding the others constant (Minetti, 1994; Herzog, 2014; Proske et al., 2014; Herzog, 2018). However, during natural human movements, muscle length and force typically change simultaneously, and their interaction may shape nonlinear effects in yet unexplored ways (Kostyukov et al., 2022; Gorkovenko et al., 2025). Whether hysteresis depends solely on the direction of length change or is also modified by the direction of force change remains unclear. This question has remained largely inaccessible due to methodological constraints.

To address this limitation, we recently developed an experimental approach enabling independent control of changes in muscle length and force during human arm movements. Using a robotic mechatronic device (RMD), externally imposed planar hand movements can be produced while participants voluntarily generate force via a visual biofeedback method. Muscle length and force trajectories are identified using methods based on individualized biomechanical modeling of bones and muscles. This approach has already been applied to circular two-joint movements and to linear cyclic changes in elbow flexor length combined with controlled force generation (Kostyukov et al., 2022; Gorkovenko et al., 2025). Whether hysteresis depends solely on the direction of length change or is also modified by the direction of force change remains unclear. This question has remained largely inaccessible due to methodological constraints.

Based on previous research, we hypothesized that muscle hysteresis is not simply determined by the direction of muscle length change (shortening vs. lengthening) or by the activation level but may also depend critically on the relationship between simultaneous changes in muscle length and muscle force. In particular, decreases in force during lengthening may attenuate hysteresis, whereas increases in force during shortening may amplify it. Thus, coinciding versus opposing directions of length and force changes may impose opposite effects on the muscle activation patterns of synergistic muscles.

## Materials and methods

### Participants

Ten healthy right-handed volunteers (6 males and 4 females, aged 26.6 ± 5.4 (mean ± SD) years) participated in this preliminary study. The experimental procedures were in accordance with the standards of the Committee for Biomedical Ethics of Bogomoletz Institute of Physiology, National Academy of Sciences, Kyiv, Ukraine, and with the 1964 Helsinki declaration and subsequent amendments or comparable ethical standards. Informed written consent was obtained from all participants. The experimental procedure did not exceed 1.2 h. Before the main session, participants practiced performing various tests up to ten times to become familiar with the setup and to correctly track the force command in the visual feedback mode.

### Experimental protocol

1. *Participant briefing:* Before the experiment, the purpose and procedure of the tests were explained to the participant;
2. *Familiarization session:* Training with the participant on how to perform the tests;
3. *EMG preparation:* Placement of surface EMG electrodes with visual assessment of recording quality;
4. *Maximal voluntary contractions (MVC) registration*: Recording of MVC for the investigated muscles;
5. *Main experimental session:* The main session consisted of 10 test conditions, each including 10 trials. Each trials lasted approximately 1 min, with rest intervals of 10–15 s between consecutive trials.

### EMG recording and data processing

Surface EMG was recorded from the following muscles: *m. biceps brachii caput longum* (*BBcl*), m. *biceps brachii caput breve* (*BBcb*), *m. brachioradialis* (*Br*), *m. triceps brachii caput laterale* (*TBclat*), and *m. triceps brachii caput longum* (*TBcl*); the abbreviations used in the paper are given in brackets. The EMG signals were recorded by pairs of electrodes (Biopac System EL 503, United States). To ensure reliable EMG recording, electrodes were placed over the belly of each target muscle parallel to the fiber direction after skin preparation (alcohol and abrasive) and palpation during voluntary contractions, with a fixed interelectrode distance of 20 mm in accordance of SENIAM recommendations (Hermens et al., 2000). Electrodes were positioned to minimize crosstalk from adjacent muscles. The recorded activities were amplified via a 16-channel amplifier (CWE, Inc., PA 19003 United States) with 10–5,000 Hz bandwidth for each channel. The following low-pass 10 Hz filtering attenuated distant interference, while preliminary movement tests confirmed discriminable EMG patterns for each muscle. The EMGs together with the position signals from the RMD were collected via a CED Power 1401 (Cambridge Electronic Design, United Kingdom) data acquisition system using the Spike 2 (v. 5.05) software (Cambridge Electronic Design, United Kingdom). The signals were digitized at 2,000 Hz, and software OriginPro 18 (OriginLab Corporation, United States) was used for off-line data analysis. The EMG signals were fully rectified and filtered (Butterworth filter of 4th order without a time shift and low-pass 10 Hz filtering) in an off-line regimen. All tests were repeated 10 times to obtain the average corresponding records. At the beginning of each experiment, we registered the maximal voluntary contraction (MVC) of each muscle undergoing study. For this purpose, the average EMG signals during steady-state maximal isometric contractions of the muscles were recorded when the shoulder and elbow angles were near 70° and 90°, respectively. The MVC tests consisted of two–three maximal isometric muscle contractions lasting 20 s, which began after a short rest period of 4–5 min after the end of the main tests. Similarly, the minimal levels of EMG activity in fully relaxed muscles were evaluated. The average EMG activity registered in the main part of the experiments is shown as a percentage scale, which ranges from the above-defined minimal level of activity (0%) to the MVCs (100%).

### Forced movements using RMD

**FIGURE 1:**
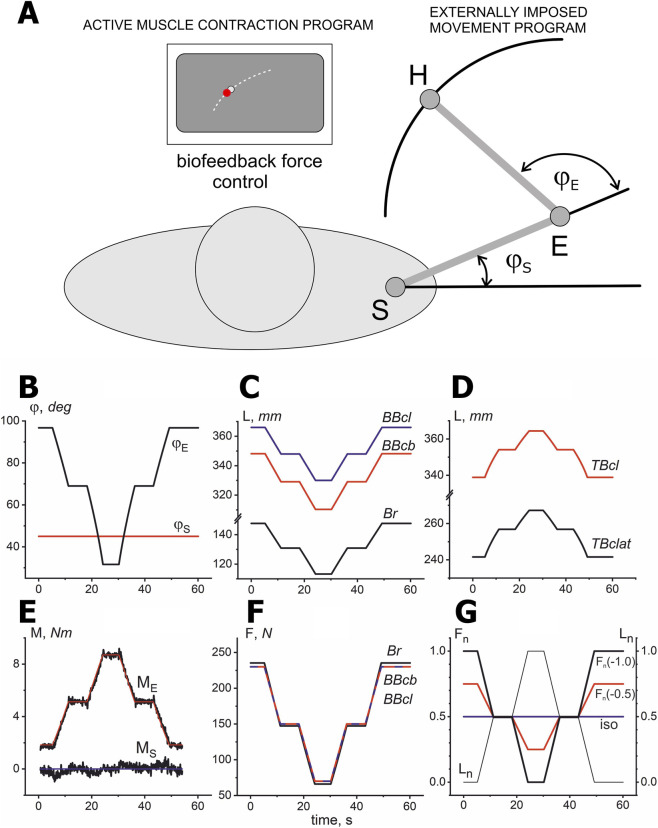
Schematic presentation of the experimental procedure **(A)**; kinematic **(B–D)** and dynamic **(E–G)** components of movement tests, which are elements of two independent programs that ensure, respectively, the externally imposed movements and the generation of forces in the biological feedback mode. **(A)** Scheme of the experiments based on separation of active (biofeedback) and passive (externally imposed) movement programs. Subjects are required to match one point on the monitor screen, which coincides with the end of the force vector, with another point representing the command signal. Passive program of elbow movements with a fixed shoulder, which is provided by RMD. **(B)** Changes in the angles of the elbow (φ_E_) and shoulder (φ_S_) joints. **(C,D)** Changes in muscle length (determined using a biomechanical model of arm muscles). **(E)** Superposition of the command signal (red) and the actual torque recording (black) generated by the force-measuring element of the RMD. **(F)** Temporal changes in elbow flexion forces using biomechanical model; trajectories were identical for *BBcb* and *BBcl*, with minor differences for *Br*. **(G)** Similarity of the force and length trajectories as well as their linearities allow us introduce their normalization in order to compare EMG reactions in different synergic muscles. The time parameters of the force and length trajectories were identical and had the shape of a double trapezoid, directed upward or downward. The amplitude of the normalized changes in muscle length (L_n_) was the same in all tests, while the force amplitudes (F_n_) varied within three limits: maximum F_n_(±1.0), intermediate F_n_(±0.5), and minimum F_n_(0), coinciding with the isotonic force (*iso*). The signs in normalized forces indicate the direction of their peaks: up (+) and down (−); similar directional differentiation in L_n_ note two patterns of the test movements P1 (up) and P2 (down).

**FIGURE 2:**
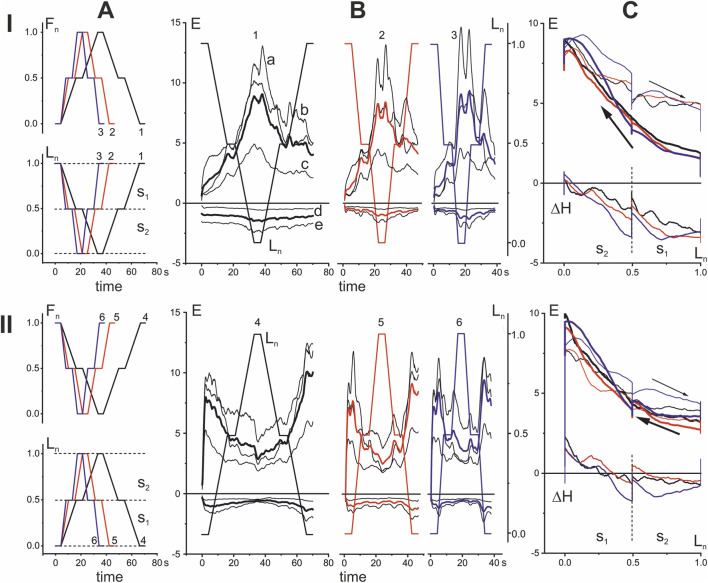
Comparison of the EMG reactions of one person for the *opposing patterns* of cyclic changes in the force and length with different velocities. **(A)** The time patterns of changes in muscle force and length are presented in normalized form (F_n_, L_n_), which schematically describe movement programs, including trajectories of the force and length changes of the muscles studied. There are shown three pairs of the force and length traces with minimal (black 1, 4), intermediate (red 2, 5), and maximal (blue 3, 6) velocities of the parameters change. Three pairs of the force and length traces have the same shape of double trapezoids, oriented upward or downward; in both cases (rows I and II) the force and lengths traces are opposite to each other. **(B)** Averaged EMG reactions recorded from three flexor (a, b, c) and two extensor (d, e) muscles (thin black lines) are shown together with their averages within each group (thick lines: black for 1, 4; red for 2, 5; and blue for 3, 6 tests). Here and on further figures, EMG records are presented in % of MVC; records are shown as positive for flexors (a–*Br*; b *BBcb*; c *BBcl*) and negative for extensors (d–*TBclat*; e *TBcl*). **(C)** Superposition of hysteresis loops E(L_n_) presents three tests with different movement velocities (1–3 in row I; 4–6 in row II). Thick and thin lines of the same color describe the “shortening” and “lengthening” branches in the loops associated with respective direction of muscle movement. The corresponding differences between the “shortening” and “lengthening” branches are shown as ΔH graphs. The negative values of ΔH correspond to the “abnormal” type of muscle hysteresis, when shortening processes require less EMG intensity compared to lengthening processes.

**FIGURE 3:**
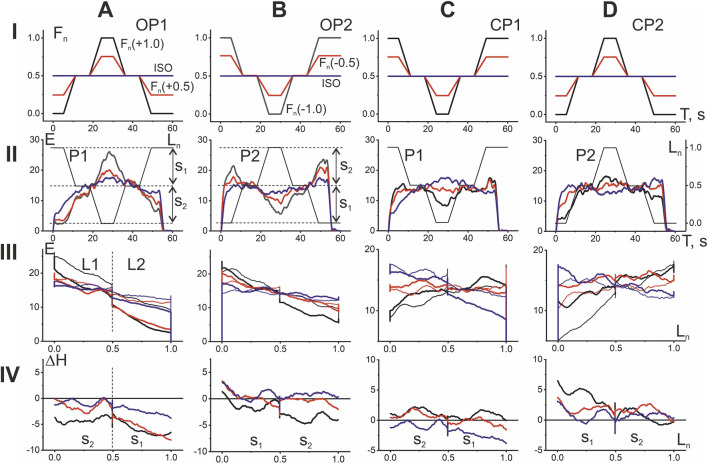
Comparison of EMG responses of elbow flexors of one person to two different *opposing* (OP1 and OP2) and *coinciding* (CP1 and CP2) *patterns* of length and force changes. The “shortening-lengthening” (P1) cycles of movements **(A,C)** and “lengthening-shortening” (P2) **(B,D)** are included in both tests, starting from the initial position of the elbow joint in an extended or bent state, respectively. In rows II and III, the averaged EMG curves of the flexors are shown by lines of the corresponding color, coinciding with the color of the F_n_ curves in row I. In addition, to better distinguish between the different EMG phases, each of the above panels also contains the L_n_ traces superimposed on it. The superposition of the hysteresis loops E(L_n_), represented in row III by thick and thin lines of the same color, describes the difference between their “shortening” and “lengthening” branches. The calculated differences between the branches are shown in row IV as the hysteresis amplitude graphs (ΔH). Sections S_1_ and S_2_ at the hysteresis and hysteresis amplitude graphs signify correspondent sectors separated by horizontal dashed lines in row II. In this and subsequent figures, sectors S_1_ always include the initial and final phases of the movement and sectors S_2_ cover their intermediate phases. In contrast to the alternation of sectors S_1_ and S_2_ associated with the movement start, division into the first and second halves of the muscle length change (L1 and L2, row III) does not vary.

It is clear that in a real single-joint movement, an experimenter can control only the joint angle changes and the force created by a participant’s hand. Earlier, it has been proposed several biomechanical models of the human arm with using magnetic resonance imaging technology of the bones of the participant’s arm with a real reproduction of their shape by 3D printing (Zasada et al., 2020). Based on such purely mechanistic models, including realistic reproduction of the shapes of real bones, muscles, and joints, fairly accurate information was obtained about changes in muscle lengths and the moments of force they create in two-joint movements (Gorkovenko et al., 2020b). In the present study, the hand motion trajectory determined by the movement of the RMD handle was programmed such that the positions of the elbow and shoulder joints did not change even without any external constraints. The accuracy of hand positioning was ±0.5 cm. At the same time, we did not also consider role of the two-joint muscle *BBcl*, which could participate in forming the torques around the shoulder joint. On the other hand, the length changes in this muscle during the test movements of the hand in the operational space were primarily considered. It has been used the previously obtained anthropometric characteristics of this muscle (Gorkovenko et al., 2020b) depending on the angle of the elbow joint, provided that the angle of the shoulder joint did not change. This allowed to convert the demanded changes in length of *BBcl* to the coordinates of the RMD manipulator with using the regression formulae for the inverse relationship between the magnitude of the elbow joint angle and the length of the muscle. The changes in the length of the *BBcb* can be closely related to such changes in the *BBcl*, so the movement trajectories of both muscles can be considered to be coincident (Figures 1–3). At the same time, due to obvious anatomical differences between the heads of the *Br* and biceps muscles, the trajectories of its movement deviate somewhat from straight lines, but due to the insignificance of these deviations, they were neglected (Figure 1C). In any case, despite the differences in the movement trajectories of different elbow flexors, the shapes of the averaged EMGs of these muscles were quite similar. On the other hand, the nonlinear distortions of the movement traces in both extensor muscles, *TBcl* and *TBclat*, were quite evident (Figure 1D).

The movement programs consisted of two types of identical double trapezoid trajectories in which the muscle length changes were directed either upward or downward; Figures 1C,D show a case of the experiment in which the length change trajectories were directed downward in flexor muscles and upward in extensors. Various combinations of the direction of change of force and length were used; the length amplitude L_n_ did not change, but forces of half amplitude F_n_(+0.5) and purely isotonic forces F_n_(0) were added to the forces of maximum amplitude F_n_(+1.0) (the plus and minus signs indicate the direction of the double trapezoid up and down) (Figure 1F).

### Active muscle contractions using biofeedback

The force occurs as a bright spot at the monitor screen signifying magnitude and direction of a force vector of the subject’s hand pressing on the RMD manipulator (Zasada et al., 2020). In parallel with the forced movement program provided by the RMD, the subject must generate a target force vector using visual feedback (Figure 1A). By focusing on this point, the subject had to align it as accurately as possible with another point, which represented a command signal for creating active force. Due to the similar anatomical properties of *BBcl* and *BBcb* regarding the generation of torque around the elbow joint, their force patterns were considered identical, while the forces generated by *Br* were estimated using the corresponding regression formulas (Gorkovenko et al., 2020a) (Figure 1D). On the other hand, in the present study we used exclusively normalized patterns of muscle forces and lengths, according to which their minimum and maximum values were taken to be equal to 0 and 1, respectively. Figure 1G demonstrates in normalized form the standard combination of three different types of the force commands (the thick lines of different color) and a single length trajectory (thin black line).

The standard double trapezoid time courses of force and length were mainly used. Both opposite (as in Figures 3A,B) and coinciding (Figures 3C,D) directions of these signals were usually used. Various combinations of the direction of change of force and length were used; the length amplitude L_n_ did not change, but forces of half amplitude F_n_(+0.5) and purely isotonic forces F_n_(0) were added to the forces of maximum amplitude F_n_(+1.0) (the plus and minus signs indicate the direction of the double trapezoid up and down). The directional variability of the test movements will be termed further as the opposing and coinciding patterns (OP and CP) in relationship between the force and muscle length changes. The lengths of the muscles during the experiment took the following values (minimum, maximum): for *BBcl* 330–366 mm, for *BBcb* 310–348 mm, for *Br* 113–148 mm. At the same time, the forces changed accordingly (minimum, maximum): for *BBcl* and *BBcb* 70–230 N (maximum force), 110–190 N (half–maximum force) and for *Br* 66–235 N (maximum force), 103–194 N (half–maximum force). With this external force, a torque of shoulder joint was 0 N. The elbow joint torque changed within 1.2–10.5 Nm (maximum force), 1.8–8.7 Nm (half–maximum force). The range of rotation of the forearm in the elbow joint was 32°–97° external angle. At the same time, the shoulder angle remained constant and its value was equal to 45°.

### Statistical analysis

For statistical evaluations, we used analysis of variance with a repeated measure (RM ANOVA) with *post hoc* Bonferroni correction. The level of statistical significance was set at p < 0.05. The specific statistical models used for analysis will be provided in the description of the respective results. IBM SPSS Statistics 26 software was used for the statistical analysis.

## Results

### *Opposing pattern* of test movements with different velocities in force and muscle length changes

For choosing an optimal parameter of tests in experiments on population of subjects we compare several identic tests with *opposing pattern* (OP) of force and length changes (Figure 2). The nature of the averaged EMG responses in the corresponding muscle populations in the compared tests looks very similar (thin black lines a, b, c–flexors and c, d–extensors in Figure 2B); the same was observed when averaging within these two sets (thick lines of different colors in Figure 2B). It can be noted that the EMG responses in the extensors were significantly weaker compared to the flexors, which indicates their auxiliary coactivation role. By comparing the hysteresis relationships between normalized muscle lengths and averaged EMGs, the influence of any important test parameter, such as movement velocity, can be seen more clearly in the averaged reactions of all flexor muscles (Figure 2C). Comparing two OP tests that differ in the initial positions of the movements (rows I and II in Figure 2), in which the movements begin from an extended or bent elbow joint and, accordingly, from the maximum and minimum initial values of L_n_, one can observe significant differences in the shape of the hysteresis loops E(L_n_). In the first case, they are noticeably wider, which is maintained for all movement velocities. Close correspondence has been also observed for the amplitude dependencies (ΔH) of the hysteresis loops in these test groups. Moreover, even visually we can judge about existence of significant differences between the two groups of tests. It can be concluded that in both cases there is a paradoxical contrast to the naturally observed predominance of the intensity of central commands in shortened muscles compared to elongated ones. First, it is well seen on higher placement of the lengthening branches in hysteresis loops E(L_n_) which are shown by thin lines in Figure 2C. Second, this is also follows from positivity of the hysteresis amplitudes ΔH presenting the difference between the shortening and lengthening branches of hysteresis loops. This is clearly seen in the higher positions of the lengthening branches of the hysteresis loops E(L_n_), shown by the thin lines in Figure 2C. On the other hand, it is clearly reflected in the negative deviations of the hysteresis amplitudes ΔH(L_n_), which represent the length-dependent distance between the shortening and lengthening branches of the hysteresis loops.

It can be assumed that the rather noticeable differences in the hysteresis effects in the two types of tests are related to the initial phases of the movements. In the first group of tests (Figure 2CI), their initial phases correspond to a more natural pattern of contraction of the flexor muscles, i.e., their flexion from the extended state of the joint. In contrast, in the second group (Figure 2CII), the initial half of the test movement consists of muscle lengthening. On the other hand, the active forces generated in the OP tests are directed in opposite directions, so this may be a more important factor that is usually taken into account when considering muscle hysteresis during movement under a constant external load. In any case, it can be argued that a “more natural” initial flexion movement under the action of changing forces is one of the possible reasons for the appearance of “more unnatural” shifts in the hysteresis amplitudes towards negative values (compare Figure 2CI,II).

### Comparison of OP and CP tests depending on initial positions and amplitudes of force change

The experiment presented in Figure 2 allowed us to select the optimal parameters of test speed and duration for comparing four possible combinations of directions with simultaneous cyclic changes in muscle forces and lengths. Figure 3 demonstrates four combinations of the force and length changes in OP and CP tests. In addition to comparing the directional sensitivity of the EMG responses, the possible influence of the initial positions of the test movements, as well as the influence of different amplitudes of force change, including constant force, are also analyzed. EMG responses are compared at three levels of force change: maximum, half-maximum, and constant or isotonic. The forces and corresponding EMG records are highlighted in the same colors–black, red, and blue, respectively. The maximal levels of the force (at apexes of double trapezoid), which were precisely evaluated for *BBcb*, did not exceed 250 N for the group of 10 subjects who participated in experiments.

The OP and CP tests are divided into two components each in respect to different initial positions of movement (Figure 3). E(L_n_) records demonstrate clear hysteresis shapes with different branch slopes relative to the first and second halves of the entire L_n_ range (L1 and L2). The exact distances between the shortening and lengthening branches of the hysteresis loops are presented in the form of graphs of the hysteresis amplitude ΔH(L_n_), which provide a clearer visual representation of the direction of the loops and the change in their width. A positive (negative) value of this parameter indicates a natural relationship between the processes of lengthening and shortening in the muscle, which require, respectively, a more intense (less intense) efferent inflow to perform a given movement. However, in the OP1 tests, a negative value for this parameter is observed even at constant forces, as reflected by the blue lines on the ΔH(L_n_) graphs, whereas in the OP2 tests, only small areas of negative values are observed. Increasing the force amplitude in the OP1 and OP2 tests leads to a negative shift in the ΔH(L_n_) lines, whereas in the CP1 and CP2 tests, these lines shift toward positive values. For a detailed comparison of hysteresis loops, it seems useful to introduce their division into sections S_1_ and S_2_, separating the initial and final quarters of the entire trace from the two intermediate ones (see rows II-IV in Figure 3). With this representation, the beginning of movements on the graphs E(L_n_) and ΔH(L_n_) is always associated with the section S_1_.

### The population averaging of the EMG responses in the group of 10 subjects

**FIGURE 4:**
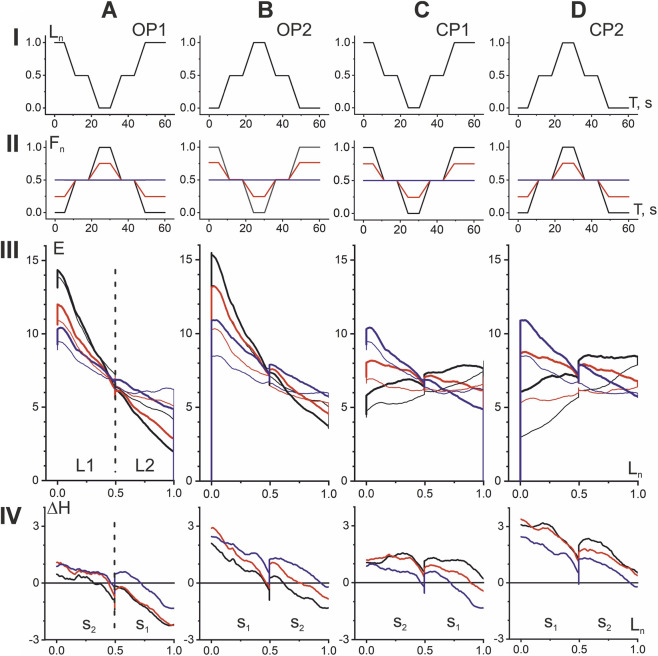
Ten-subject population averaging of the preliminary averaged EMG reactions of three flexor muscles in each person, which were recorded in standard movement tests with three levels of force amplitude. Rows I and II show the patterns of change in length and force; rows III and IV present the hysteresis loops and hysteresis amplitudes in dependence on the normalized muscle lengths; there are compared reactions **(A,B)** on two *opposing* (OP1, OP2) and **(C,D)** two *coinciding* (CP1, CP2) *patterns* of the test movements; black, red, and blue colors denote three different force amplitudes (see Figure 3). The thick and thin curves in row III represent the EMG responses during muscle shortening and lengthening, respectively.

The similarity in the nature of the standard test movements is clearly evident across the different subjects who participated in the study; this applies both to the shape of the EMG dependencies recorded from the three elbow flexors initially and after their averaging, and to the features of the hysteresis amplitude graphs described above (Figure 3). On the other hand, given the uniform nature of changes in muscle force and length across subjects, and the use of normalized muscle lengths, it seems possible to apply a population averaging procedure to the data. Initially, this procedure was applied to the averaged EMG recorded from ten subjects, and then the corresponding dependencies were constructed similarly to those for individual subjects (Figure 4).

Surprisingly, the population-averaged data in Figure 4 further highlight some important differences between the hysteresis effects in the four test movement variants. First of all, if to compare the pairs of the OP and CP tests, it can be pointed out a more extent of elongation of the hysteresis loops and hence rise of their entire amplitudes in the first pair. The slopes of the branches in the OP loops relative to L_n_ are negative in the ranges of lengths L1 and L2, being higher in absolute values in L1. Rise in the amplitude of the force changes (the line colors order: blue–red–black) evokes a rise in the branch slopes as well as downward shift of the hysteresis amplitudes (Figures 4A,B, rows III and IV). However, in OP2 tests, hysteresis effects are more pronounced, since the sets of three ΔH lines are shifted upward compared to OP1. Moreover, in OP2 tests, the area of zones with “anomalous” hysteresis in the range of L2 lengths, where the hysteresis amplitude takes negative values, becomes smaller compared to OP1. The slopes of the branches in hysteresis loops are less expressed in the both CP tests, and the loops itself are turning with rise of the force amplitude oppositely to their turning in OP tests; at high forces these slopes may become completely positive (Figures 4CIII,DIII). In contrast to the OP tests, in the CP tests, along with the opposite rotation of the hysteresis loops with an increase in the force amplitudes, an upward shift of the successive ΔH curves is also observed, so in general they are located higher compared to the OP tests. Thus, the probability of detecting zones with an “anomalous” negative direction of hysteresis effects is higher for OP tests compared to CP.

It should be noted that the transition to negative values of the hysteresis amplitude can be observed even at constant forces in tests OP1 and CP1, which in this case coincide with each other (blue lines in Figures 4AI,II,CI,II). It also seems important that population averaging confirms the earlier conclusion (see Figure 3) that increasing the amplitude of force change (moving from blue to red and black lines) causes an obvious downward shift of the ΔH curves in OP tests and an upward shift in CP tests. As a result of these shifts, the “anomalous” hysteresis reactions with negative amplitudes practically disappear in CP2 tests and increase significantly in OP1 tests, however, this concerns mainly the L2 length range, while in the L1 range the hysteresis amplitudes remain predominantly positive.

### Combining OP and CP responses to the same movement paradigms

**FIGURE 5:**
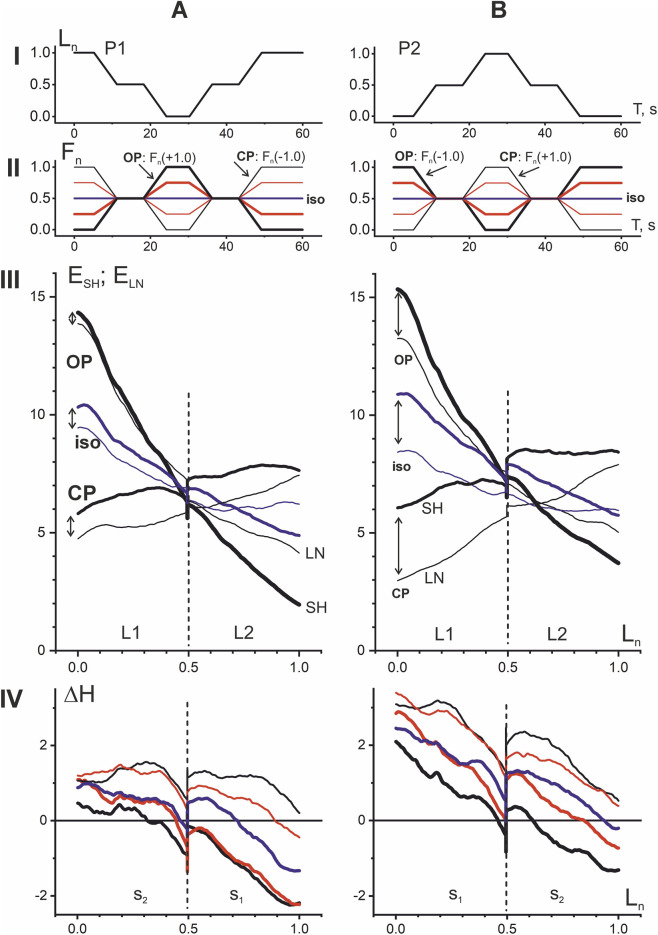
Comparison of EMG responses in P1 **(A)** and P2 **(B)** movement patterns. The four types of EMG responses, divided in Figure 4 into *opposing* (OP1, OP2) and *coinciding* (CP1, CP2) *patterns*, are combined into subgroups of identical movement patterns P1 and P2. For greater clarity, the intermediate force reactions F_n_(±0.5) are not shown in the hysteresis loops (row III), but they are present in the hysteresis amplitude graphs (row IV). The *opposing patterns* of EMG responses are shown by thicker lines (rows II, IV). Note the wider distances between the shortening and lengthening branches of hysteresis loops (marked by vertical arrows in row III), which is the reason for the overall upward shift of the hysteresis amplitude trajectories in P2 (row IV).

The above four types of EMG responses in the elbow flexor muscles show that the OP and CP tests can be compared even better if they are combined with respect to the same P1 and P2 movement types (Figure 5). In this case, the CP tests represent a natural transformation of the corresponding OP tests with a sequential shift of the F_n_ vertices downwards (upwards) in the P1 (P2) movements. When viewed in this way, both hysteresis loops and hysteresis amplitudes exhibit fairly similar dynamics of change, while also revealing significant differences. A similarity may be pointed out in respect of turnings of the hysteresis branches in L1 and L2 length ranges. In P1 and P2 movement patterns, these branches rotate counterclockwise around a small region at the boundary of the L1 and L2 length ranges. This rotation causes the left and right parts of the hysteresis loops to move in opposite directions—downward (upward) within the L1 (L2) length ranges. As for differences, note the greater distance between the shortening and lengthening branches of the hysteresis loops, indicated by vertical arrows in Figures 5AIII,BIII. This explains the overall upward shift in the hysteresis amplitude trajectories in the P2 tests (row IV in Figure 5). Despite the similarity of the ΔH trajectories in the P1 and P2 tests, which concerns, their noticeable downward shift towards muscle lengthening, as well as a fairly rigid alternation within the used order of F_n_ changes (see the colors of the curves), the general upward shift of the ΔH trajectories in the P2 tests seems important.

### Preliminary quantitative analysis of the EMG hysteresis effects during cyclic movements (population averaging)

**FIGURE 6:**
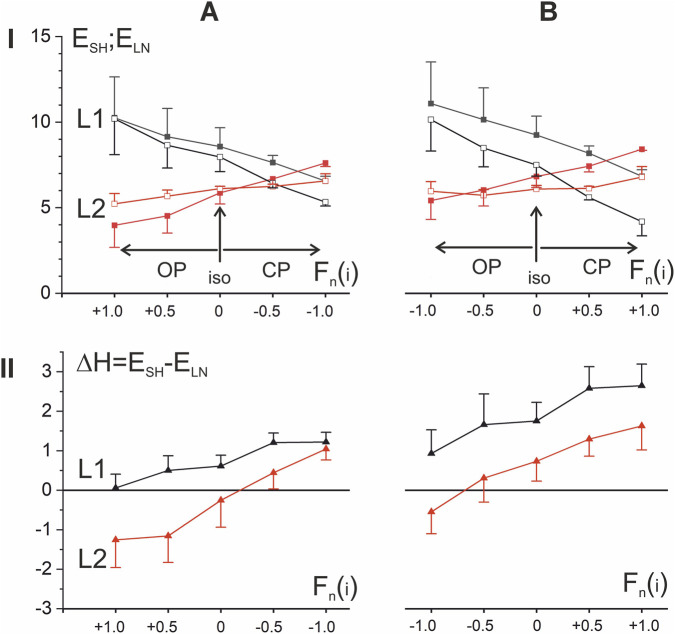
Quantitative analysis of the ten-subject population averaged EMG reactions presented in Figures 4, 5. **(A,B)** Signal segments corresponding to regions of linear change in length and force were extracted from the averaged EMG recordings (*N* = 10 participants), then a standard statistical procedure was applied to the sets of signal points belonging to these segments (n = 600) to determine their mean ± SE (row I). The black and red dots correspond to the EMG parameters recorded in the first (L1) and second (L2) halves of the muscle length change; the closed and open symbols in row I correspond to the shortening (SH) and lengthening (LN) phases of the movement. Of the five sets of dots in rows I and II, the middle sets correspond to isotonic movements; the data relating to *opposing patterns* are directed to the left, and *coinciding patterns* to the right. The statistical evaluation of hysteresis amplitudes (row II) was determined after preliminary subtraction of EMG lengthening records from the corresponding shortening curves; a negative value of this parameter indicates a violation of the natural direction of muscle hysteresis. Each point represents the mean ± SEM.

For a simplified quantitative description of the population-averaged EMG responses of the ten subjects shown in Figures 4, 5, we applied the standard statistical procedure (mean ± SE) directly to the points constituting the corresponding sections of the EMG loop (Figure 6), so the results obtained can only be used for a preliminary description of the general trends in the data; a detailed statistical analysis of the corresponding EMGs in the subject group is presented in the next paragraph. In any case, the obtained results provide an idea of the EMG intensity in the shortening and lengthening parts of the test movements, as well as the tendencies of their changes when the amplitude of force changes in both directions compared to the isotonic mode. It appears that one of the most interesting conclusions that can be drawn from comparing the dependences of the mean values of the EMG responses on the force amplitudes is the opposite direction of their changes in the L1 and L2 length ranges (compare the black and red lines in Figures 6AI,BI). Regarding isotonic data, a change in force amplitude in the OP (CP) direction causes an increase (decrease) in EMG intensity in the L1 range, while in the L2 range such reactions are opposite. Another important difference between the EMG responses in different length ranges is the appearance of “anomalous” hysteresis amplitudes in the L2 range (negative values of the ΔH dependencies in Figures 6AII,BII). On the other hand, a common upward shift of these curves diminishes noticeably these effects for P2 movement patterns (Figure 6BII).

### Statistical analysis of the EMG hysteresis effects during cyclic movements

To assess the repeatability of EMG signals and the magnitude of the generated effort, we performed the corresponding Intraclass Correlation Coefficient (ICC) tests. Such tests were performed for all participants (10), for all tests (10) and for the muscles under study (5). Accordingly, a total of 500 ICC tests were performed using the ICC(2, 10) (Two-Way Random, Average Measurement) scheme. The lowest value was ICC(2, 10) = 0.693. From which we concluded that the level of reproducibility of the registered signals had moderate reliability.

A *post hoc* power analysis was conducted (α = 0.05, *N* = 10), which indicated that the observed power for significant factors and their interactions is mostly 0.50–0.95 as one could see in Tables 1 and 2. Therefore, the present sample reliably detects large effects, and smaller effects should be considered preliminary.

**TABLE 1:** Statistical assessment of the influence of experimental conditions on the EMG intensity recorded in the elbow flexor muscles (see Figures 7A–C).

Factors	F	p	Sig	η^2^_p_	OP
PMOV	6.920	0.027	*	0.435	0.650
FDIR	16.942	<0.001	**	0.653	1.000
MDIR	5.001	0.052		0.357	0.514
RLEN	39.480	<0.001	**	0.814	1.000
PMOV × FDIR	1.868	0.137		0.172	0.510
PMOV × MDIR	66.546	<0.001	**	0.881	1.000
FDIR × MDIR	5.386	0.002	**	0.374	0.953
PMOV × FDIR × MDIR	14.052	<0.001	**	0.610	1.000
PMOV × RLEN	4.883	0.054		0.352	0.505
FDIR × RLEN	42.152	<0.001	**	0.824	1.000
PMOV × FDIR × RLEN	1.832	0.144		0.169	0.501
MDIR × RLEN	27.305	0.001	**	0.752	0.996
PMOV × MDIR × RLEN	3.159	0.109		0.260	0.356
FDIR × MDIR × RLEN	3.889	0.010	*	0.302	0.856
PMOV × FDIR × MDIR × RLEN	1.725	0.166		0.161	0.475

The table presents the results of the 4-factor RM-ANOVA; the cross represents the interaction of factors; the last column indicates the significance of the result. One and two asterisks correspond to p < 0.05 and p < 0.005, respectively; OP, observed power; η^2^_p_–partial eta squared.

**TABLE 2:** Statistical assessment of the influence of experimental conditions on difference between movement directions (see Figure 7D).

Factors	F	p	Sig.	η^2^	OP
PMOV	19.962	0.002	**	0.689	0.977
FDIR	14.206	<0.001	**	0.612	1.000
RLEN	30.465	<0.001	**	0.772	0.998
PMOV × FDIR	0.732	0.576		0.075	0.213
PMOV × RLEN	2.639	0.139		0.227	0.306
FDIR × RLEN	2.658	0.048	*	0.228	0.682
PMOV × FDIR × RLEN	0.998	0.422		0.100	0.283

The table presents the results of the 4-factor RM-ANOVA; the cross represents the interaction of factors; **Sig. column** indicates the significance of the result, one and two asterisks correspond to p < 0.05 and p < 0.005, respectively; OP, observed power; η^2^_p_–partial eta squared.

To get quantitative information about the main characteristics of the EMG hysteresis during cyclic movements and their change in dependence on parameters of the test movements, the mean EMG values at different stages of the movement program were defined in ten subjects participating in the present experiments. There were four such stages, corresponding to time intervals during which muscle length changed–either shortening or lengthening. Thus, for each motor task, four mean EMG values (M1, M2, M3, and M4) were obtained to evaluate hysteresis magnitude.

Subsequently, each value was assigned a specific code according to the mechanical parameters of the test and its position within the task’s time interval, in order to enable further statistical analysis. In this way, each level of each factor was given a special designation. We distinguished four factors: movement pattern (PMOV), possessing two levels P1 and P2, range of length change (RLEN) with two levels: L1 and L2, force direction (FDIR) with five levels: OP1, OP2, ISO, CP2, CP1, and direction of the muscle length change (MDIR) with two levels: LN and SH.

Accordingly, four values with the corresponding codes were obtained for each test. For example, in the first test (OP1) the muscle initially shortened (movement phases M1 and M2) and subsequently lengthened (phases M3 and M4). During this movement program, the muscles created force that increased during phases M1 and M2 and decreased during phases M3 and M4. Thus, the values were coded as follows:

- M1 – P1, L2, OP1, SH
- M2 – P1, L1, OP1, SH
- M3 – P1, L1, OP1, LN
- M4 – P1, L2, OP1, LN

It should be noted that such values (M1–M4) with their respective coding were obtained for all participants in the framework of standard test program; for each participant, a standard data table consisting of 40 rows had been generated. Subsequently, the data from all 10 participants were recombined into a final table of 400 rows, which was further analyzed using the SPSS software (IBM SPSS Statistics version 26 (IBM Corp., Armonk, NY, United States). For statistical analysis, we applied repeated-measures analysis of variance (RM ANOVA) followed by *post hoc* Bonferroni tests; statistical significance of results had been defined at the level p < 0.05. The effects of experimental conditions on the obtained results were assessed using three-way (3-way, Table 2) and four-way (4-way, Table 1) RM ANOVA. Post hoc analyses were performed using the Bonferroni test.

**FIGURE 7:**
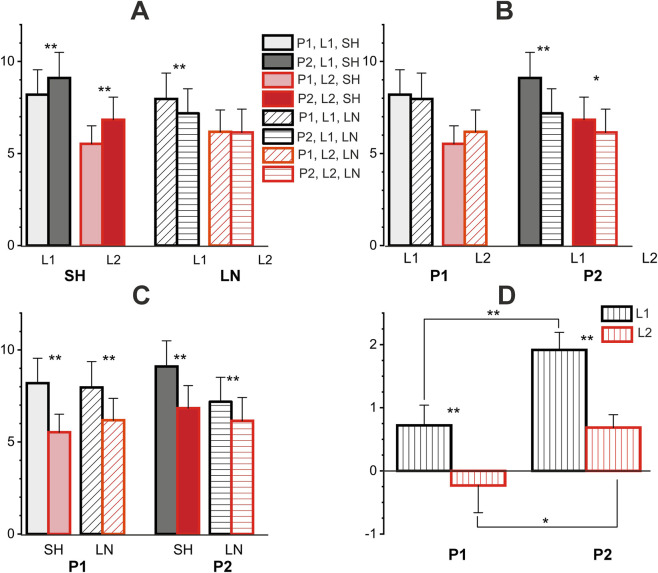
Statistical pairwise comparisons between various factors influencing EMG intensity in test movements. **(A–C)** pairwise comparisons of different factors, where each panel represents the same columns with a different permutation for comparison. **(D)** Pairwise comparisons of differences in EMG responses corresponding to oppositely directed movements. P1, P2 – patterns of test movements; L1, L2 – first and second ranges of muscle length change; SH, LN–muscle shortening and lengthening. One and two asterisks correspond to p < 0.05 and p < 0.005, respectively. Each point represents the average value (*N* = 10 participants) in form mean ± SEM.

From the above statistical analysis, it can be seen that the movement pattern factor has highly statistically significant influence on EMG indices (see Tables 1 and 2; Figure 7A). This can be explained by the fact that the EMG parameters of the activity of the corresponding muscles, including the hysteresis parameters, are significantly influenced by the length of the muscle at which the movement begins. Thus, for the P1 and P2 patterns, the difference is precisely that in the first case the length of the corresponding elbow flexors was close to the maximum, and in the second to the minimum, respectively. Also, the factor of range of the muscle length (with levels L1 or L2) had a high influence on the EMG parameters (see Tables 1 and 2; Figures 6, 7C). Thus, for all cases, the corresponding values for L1 were higher than for L2. The variation in forearm and upper arm flexor activity at different elbow angles (and, consequently, different muscle length) can be attributed to changes in both mechanical and neural factors. As the joint angle alters, muscle length and the flexor moment arm vary, influencing torque production. At extreme flexion or extension, suboptimal overlap of actin and myosin filaments reduces contractile efficiency, requiring greater EMG activation to maintain the same load. At the same time, the factor of muscle length change direction–lengthening or shortening–did not reach the level of statistical significance, although it was close to it (p = 0.052). However, a high level of interaction of the factors MDIR × RLEN was noted (p = 0.001, Table 1). Thus, in Figure 7B, it can be seen that the corresponding EMG characteristics were higher under conditions of muscle shortening than during muscle lengthening (only in the case of movement pattern P2, i.e., lengthening–shortening). This can also be explained, at least in part, by the different initial conditions of the beginning of muscle movement. This also explains why the level of hysteresis is higher precisely in conditions of movement pattern P2 (see Figures 6AII,BII, 7D).

## Discussion

Any purposeful movement of the human arm involves multiple synergistic muscles performing various functional roles, one of which may be primary. In other motor tasks, these muscles may switch to auxiliary or counteracting functions, requiring a natural redistribution of incoming central motor commands between the muscles. The intensity of efferent muscle activation, which might be assessed by EMG signals, may depend on the forces acting on the muscles and the actual change in their length. In real movements, these signals can also be significantly influenced by the nonlinear dynamic properties of muscle contractions, one of which is muscle hysteresis. Hysteresis effects have also been demonstrated in movements involving multiple joints. Brown et al. (2022) found that hysteresis was greater in the group with chronic ankle instability and hypermobility than in the group without hypermobility. Changes in tissue properties in the group with chronic ankle instability and hypermobility likely indicate a reduced ability of the lateral ankle complex to respond to loading.

Traditionally, the muscle hysteresis is often treating via differences in forces generated by the constantly activated muscle in concentric, eccentric, and isometric contractions; the concentric and eccentric types of contraction correspond to the shortening and lengthening movements. The hysteresis is forming even during very slow movements, it also appears in isometry under reciprocating changes in the rate of the arriving efferent activity, and possesses by steady after-effects (Kostyukov, 1998). One of the main problems for a real assessment of the effects of hysteresis in the formation of real patterns of EMG activity entering muscles under conditions of movement is the limitation of our knowledge about muscle hysteresis itself. The muscle hysteresis is a fundamentally nonlinear process, which, like most of such processes, does not lend itself to precise mathematical description. An analytical description of muscle movements, taking into account hysteresis effects, can be achieved using a model consisting of a sequential connection of nonlinear static and linear dynamic elements; however, this approach is only valid for shortening processes, while muscle lengthening is significantly more complex (Proske and Gandevia, 2018; Herzog, 2018). Moreover, it is likely that the vast majority of studies analyzing hysteresis effects in isolated muscles or muscle fibers have used predominantly constant activation intensities, whereas the known experiments with modulated stimulation rates have been conducted predominantly under isometric or isotonic conditions (Herzog, 2018).

The present experiments are devoted of obtaining information about the central motor commands transmitted to muscles during simultaneous changes in both their length and force. This approach recently has been applied in our previous study (Gorkovenko et al., 2025), in which with using a similar methodical approach there are compared opposing and coinciding elbow joint movements, however the study was restricted by using a single movement pattern, in which test movements started only from the extended elbow joint angle.

In the present study two combinations of *opposing* and two combinations of *coinciding* movement patterns with different relationships between force and muscle length changes are compared (Figures 3 and 4), additionally the EMG reactions for varying forces are compared with isotonic forces. Such a variety in the force and length input changes allows to unify the registered EMG reactions within two group of the shared responses presenting a continuous transition from *opposing* to *coinciding* movement patterns via an intermediate regime of isotonic contractions (Figure 5). This approach create possibility for identification of essential differences between EMG reactions in different zones of the length change (L1, L2), as well as determination of opposite directions of their change for opposing to *coinciding* movement patterns (Figure 6).

Using identical, both *coinciding* and *opposing*, time patterns of cyclic changes in the length and force of the muscles, we showed, firstly, that the amplitude of the EMG hysteresis apparently depends on the ratio of the amplitudes of these parameters and, secondly, with an increase in the ratio, this amplitude increases in the first movement pattern, while in the second it clearly decreases, even reaching negative values (Figure 5). The overall trend in hysteresis amplitude changes demonstrates a marked decrease with increasing muscle length; at maximum length values, some trajectories become negative, indicating a disruption of the natural shape of hysteresis loops in active muscles. On the other hand, these records confirm a strict orderliness, with the trajectories of maximum force amplitudes in *opposing* tests always being at the bottom, while subsequent steps in force amplitude continuously shift these trajectories upward.

For two halves of the length range, a difference in the direction of change in EMG amplitudes plotted against the 5-point set of force amplitudes was unexpectedly observed (row in Figure 6). Although the hysteresis amplitudes change similarly on the graphs plotted in respect to a step-wise transition of the force amplitudes between OP to CP movement patterns, directions of change of the correspondent EMG amplitudes were different (compare rows II and I in Figure 6). Along with these changes, one can also note the reverse order of the arrangement of parts of the shortening and lengthening branches in the corresponding dependencies, which ultimately leads to the inversion of the “normal” direction of the hysteresis loops.

It seems to be also important to stress existence of differences in influence of two different patterns of the test movements (P1, P2) on the studied dependencies of EMG intensity, and the hysteresis amplitude. This applies both to the population-averaged curves (Figures 5 and 6) and to statistical pairwise comparisons between various factors influencing EMG intensity in the studied group of subjects (Figure 7; Tables 1 and 2). The population-averaged EMG responses and corresponding hysteresis amplitudes are clearly shifted upward in P2 trials compared to P1 trials (Figures 6A,B), and pairwise comparisons in this case show a statistically significant difference in this factor within the group of shortened responses in both the L1 and L2 ranges, as well as between shortened responses in the L1 range (Figure 7A). At the same time, all movement tests used in the present study, which are represented by Figures 5A,B, could be considered as completely coincident when focusing attention only at phases of movement. Indeed, the difference between the two sets of length and force trajectories in tests P1 and P2 lies only in the order of alternation of two halves of the test movement, each containing two successive phases of muscle shortening or lengthening, but the statistical analysis applied considers the results concerning both halves. Perhaps the seemingly strange difference in the results of the P1 and P2 tests is explained solely by the completely different initial conditions for each half of the test. In any case, the present study likely supports the notion that both the initial conditions of movements and their after-effects can play a significant role in all processes mediated by the muscular and neural elements of the motor control system, each of which is known to exhibit pronounced hysteresis properties (Yeo et al., 2023; Proske, 2023; Proske and Gandevia, 2018; Proske et al., 2014; Herzog, 2018; Pinnell et al., 2019).

The apparent dependence of hysteresis effects in muscle contractions demonstrated in the present study with relatively weak activation of a limited number of arm muscles may also likely play a role in generating motor commands to synergist muscles involved in more intense motor activity. Movement- and activation-dependent processes involved in the modification of muscle hysteresis appear to need to be considered, particularly in movement programs with a high degree of antagonist muscle involvement, such as walking and running, in which coactivation and reciprocal activation of antagonist muscles are of primary importance (Gorkovenko et al., 2012; Minetti, 1994). In multijoint movements or highly coordinated movements, including movements of the entire body and several limbs, such as walking, three partially independent types of muscle synergy are usually considered: kinematic synergy (Santello and Soechting, 2000; Thakur et al., 2008; Soechting and Flanders, 1997), force (kinetic) synergy (Santello and Soechting, 2000; Grinyagin et al., 2005), and activation (muscle) synergy (Weiss and Flanders, 2004; Castellini and van der Smagt, 2013; Valero-Cuevas, 2016; Latash et al., 2007; Bruton and O'Dwyer, 2018; Ivanenko et al., 2004; Wang et al., 2013; Valero-Cuevas, 2000).

Currently, in the community of researchers studying various problems associated with nonlinear effects in the dynamics of muscle fiber contractions, the opinion often dominates that the cross-bridge theory, although it explains well the mechanical properties of isometric and concentric contractions, poorly explains the mechanics of eccentric contractions and the increase in residual force enhancement associated with them (Herzog, 2018; de Campos et al., 2022). To date, the most widely accepted and long-standing proposal to explain residual force enhancement is the sarcomere length heterogeneity theory (Brown et al., 2022; Zasada et al., 2020). Based on the available data, titin appears to be one of promising candidates for explaining the mechanisms underlying residual force enhancement, while instability and sarcomere length heterogeneity are likely not involved in these effects (Tomalka, 2023; Millard et al., 2024; Herzog, 2014). Titin is a giant sarcomere spring that stabilizes filaments, provides passive force, and participates in active force production by binding to actin during eccentric contractions, thereby increasing stiffness and contributing to residual force enhancement. Titin is a spring capable of adapting its stiffness depending on crossbridge activation and strength, allowing it to provide an excellent explanation for residual force enhancement without changing the core principles of crossbridge theory (Julian and Morgan, 1979; Morgan, 1994). The biomechanical principles underlying muscle hysteresis may be relevant for both normal neuromuscular function and surgical reconstruction, and are also important for research on physical activity in athletes (Kuliś and Gajewski, 2022; Migliorini et al., 2024b).

Our study demonstrates that an adequate understanding of the hysteresis effects associated with the residual force enhancement during controlled muscle lengthening is likely fundamentally insufficient without a comprehensive, combined analysis of the lengthening and shortening processes. Furthermore, limiting such experimental approaches to a constant level of muscle activation may lead to misleading conclusions, as muscle hysteresis can vary significantly depending on the relative direction of change in both muscle length and activation intensity. The present results demonstrate that the direction and magnitude of force changes relative to length changes can substantially modulate EMG hysteresis, revealing nonlinear effects that were previously unexplored.

These findings also have clear practical implications. In neuromuscular rehabilitation, the insights gained can inform more effective training and retraining of coordinated arm movements in patients recovering from injury or neurological disorders. In robot-assisted therapy, the results can guide the development of exoskeletons or assistive devices that better adapt to the nonlinear characteristics of human muscle contractions, optimizing force and motion trajectories for safer and more efficient therapy. In sports biomechanics, knowledge of hysteresis behavior during different movement patterns can inform the design of training programs aimed at improving performance and reducing injury risk in athletes performing rapid or cyclic arm movements.

### Practical implications

- Neuromuscular rehabilitation: Understanding how force-length interactions affect muscle activation patterns can help optimize rehabilitation protocols for patients recovering from injury or stroke, ensuring more effective retraining of coordinated movements.
- Robot-assisted therapy: The insights into hysteresis effects can inform the design of robotic exoskeletons or assistive devices, allowing them to better adapt force and motion trajectories to human muscle nonlinearities for safer and more efficient therapy.
- Sports biomechanics: Knowledge of how muscle hysteresis varies with different movement patterns can guide training programs aimed at improving performance or preventing injury, particularly in sports involving rapid or cyclic arm movements.

### Limitations and future work

This study has several limitations that should be acknowledged. First, the sample size was relatively small (*N* = 10), which limits the statistical power for detecting medium or small effects and may restrict generalizability. Second, the experiments were conducted on a limited number of elbow flexor muscles and specific movement patterns; thus, the findings may not fully extrapolate to other muscles, joints, or multi-joint tasks. Third, while we examined EMG hysteresis under controlled laboratory conditions, real-world movements involve additional complexity, such as variable joint loading, muscle fatigue, and interaction with external environments. Finally, the study focused on short-term measurements; longer-term adaptations and learning effects were not assessed.

Future studies should include larger, more diverse participant cohorts to improve statistical power and generalizability. Additionally, exploring the effects of fatigue, motor learning, and patient populations (e.g., neurological or musculoskeletal disorders) would help translate these findings to neuromuscular rehabilitation. Integration with robot-assisted therapy and sports biomechanics applications could provide practical recommendations for optimizing movement training and assistive device design.

## Conclusion

The present study demonstrates that muscle hysteresis is strongly influenced by the interaction between muscle length and force changes, showing that the EMG responses of elbow flexor muscles are critically dependent not only on the direction of these changes but also on the relative amplitude of the force variation. These findings indicate that the same pattern of muscle length change can produce markedly different EMG hysteresis responses depending on whether the accompanying force changes coincide with or oppose the movement. Furthermore, the results highlight that prior movement history plays a key role in shaping muscle activation patterns, emphasizing that the effects of previous muscle activity can alter the magnitude and direction of subsequent EMG responses. This underscores the importance of considering both the dynamic interplay of mechanical factors and the history-dependent properties of muscles when interpreting neuromuscular behavior during cyclic or repetitive movements.

## Data Availability

The datasets presented in this article are not readily available because of Local law. Requests to access the datasets should be directed to kostyuko@biph.kiev.ua.
